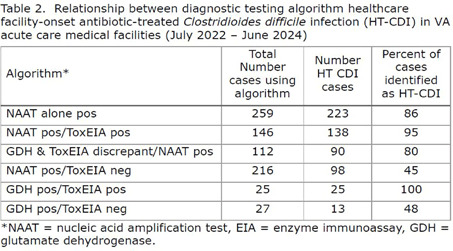# The Impact of a Healthcare Facility-Onset Antibiotic-Treated CDI Surveillance Definition on reportable cases in VA Medical Centers

**DOI:** 10.1017/ash.2025.189

**Published:** 2025-09-24

**Authors:** Brian McCauley, Martin Evans, Natalie Hicks, Loretta Simbartl

**Affiliations:** 1 Department of Veterans Affairs; 2 University of Kentucky School of Medicine/VHA; 3 National Infectious Diseases Service (NIDS); 4 Department of Veterans Affairs

## Abstract

**Background:** Most laboratories diagnose Clostridioides difficile infection (CDI), using a nucleic acid amplification test (NAAT) or an enzyme immunoassay (EIA) for detecting the toxins in the stool. Adoption of a two-step testing method where a positive NAAT is folowed by a negative toxin EIA may lead to underreporting of patients with CDI healthcare-associated infections (HAIs). The possibility that patients with an active infection may be underreported has been suggested by data, that NAAT positive/Toxin negative patients are sometimes treated. Therefore a new surveillance definition has been adopted by NHSN, healthcare facility-onset antibiotic-treated C. difficile infection (HT-CDI). To date, the impact of adopting this new metric on the number of CDI cases that would qualify for HAI surveillance reporting remains unknown. We chose to explore this by retrospectively evaluating CDI cases using the current and proposed HT-CDI surveillance definition over a two year period in a sample of VA Medical Centers. **Method:** The above HT-CDI definition was applied to all acute care inpatients tested for CDI from July 1, 2022 through June 20, 2024 in 25 volunteer acute care VA medical centers. Centers were chosen based on the use of NAAT alone, NAAT followed by toxin EIA, GDH followed by toxin EIA with discordant results arbitrated by NAAT, GDH plus toxin EIA, toxin EIA alone, or GDH plus NAAT. A HT-CDI was defined according to NHSN as any qualifying C. difficile-positive asssay collected in an inpatient location on day 4 or greater after admission, along with administration of new qualifying antimicrobial therapy in a time frame window. Qualifying therapeutic agents for CDI were defined as enteral vancomycin, metronidazole, or fidaxomycin or intravenous metronidazole. **Results:** Analysis of the 789 CDI cases in 25 VA facilities collected over two years showed that adoption of the HT-CDI definition could potentially increase the number of reportable cases by 23.3%. The impact was most pronounced for patients diagnosed with two-step testing where presumably the patient would not be reported to a national database on the last test done. **Conclusion:** Application of the HT-CDI definition to Veterans Affairs patients could lead to an estimated 23% increase in the number of cases identified and eligible for reporting to a national database such as NHSN. These cases may be a better indicator of the true burden of CDI in an acute healthcare system.

**Figure:**
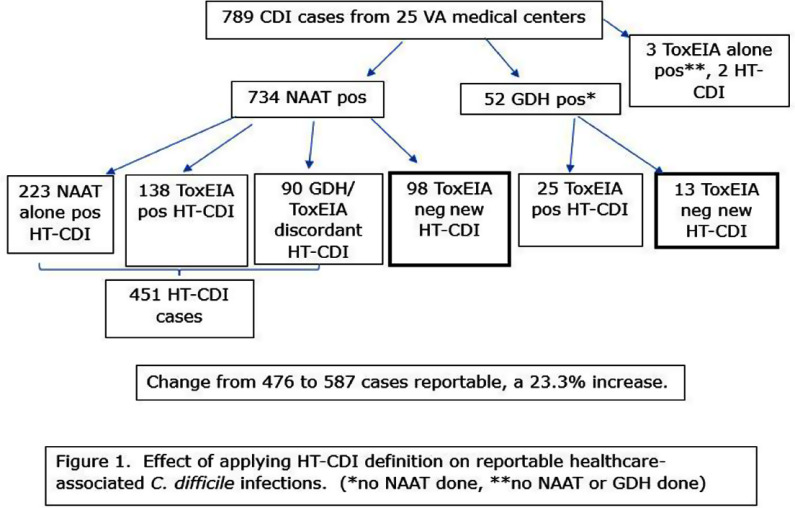

**Figure:**